# Semaglutide, Other GLP-1 Receptor Agonists, and Papillary Thyroid Carcinoma: A Narrative Review of Risk, Progression, and Mechanisms

**DOI:** 10.3390/jcm15207834

**Published:** 2026-10-11

**Authors:** Luiza Barseghyan, Iqbal Munir, Steven Chrysafide, Alfred A. Simental, Salma Khan

**Affiliations:** 1 Center for Health Disparities and Molecular Medicine, School of Medicine, Loma Linda University, Loma Linda, CA 92350, USA; 2 Department of Endocrinology, Riverside University Health System, Moreno Valley, CA 92555, USA; 3 Department of Otolaryngology–Head and Neck Surgery, Loma Linda University, Loma Linda, CA 92350, USA

**Keywords:** semaglutide, Ozempic, GLP-1 receptor agonist, papillary thyroid carcinoma, thyroid neoplasms, disease progression, pharmacovigilance, medullary thyroid carcinoma, sex differences, differentiated thyroid cancer

## Abstract

Semaglutide, a glucagon-like peptide-1 receptor agonist (GLP-1RA), is widely prescribed for type 2 diabetes and obesity. Rodent studies showed thyroid C-cell tumors, prompting a boxed warning for medullary thyroid carcinoma (MTC); whether this extends to papillary thyroid carcinoma (PTC), a follicular-cell-derived tumor, is uncertain. This narrative review examines semaglutide and other GLP-1RAs in relation to PTC, distinguishing semaglutide-specific from class-level evidence, and PTC from differentiated thyroid cancer (DTC), MTC, and thyroid cancer overall. Most human studies evaluated GLP-1RAs as a class and reported thyroid cancer overall; semaglutide-specific and PTC-specific estimates are scarce. Pooled trials and national cohorts mostly showed no statistically significant increase in thyroid cancer, although three separate meta-analyses of randomized trials each reported a statistically significant increase in overall thyroid cancer, and case-control and spontaneous-reporting studies reported signals vulnerable to detection bias and confounding. In a matched cohort of 1072 patients with DTC, GLP-1RA exposure (median 16 months) was not significantly associated with progression over 69 months. Receptor-expression findings were inconsistent, and preclinical data did not show proliferative effects. The cohort and progression findings indicate no statistically detectable increase within the available sample sizes, exposure durations, and event numbers; they do not establish oncological safety. Semaglutide-specific, histology-resolved studies with longer exposure are needed.

## 1. Introduction

Glucagon-like peptide 1 receptor agonists (GLP-1RAs), including semaglutide, liraglutide, dulaglutide, and exenatide, and the dual glucose-dependent insulinotropic polypeptide (GIP)/GLP-1 agonist tirzepatide have changed the pharmacologic management of type 2 diabetes and obesity. Semaglutide is marketed as Ozempic for diabetes and Wegovy for weight management. A U.S. retail-pharmacy analysis documented a 442% increase in monthly semaglutide prescription fills, from 471,876 in January 2021 to 2,555,308 in December 2023 [1]. This scale of exposure has increased scrutiny of rare adverse events that were difficult to detect in trials conducted before approval.

Concern about thyroid malignancy originates in preclinical toxicology. Rodent studies found increases in thyroid C-cell hyperplasia, C-cell adenomas, and medullary thyroid carcinoma (MTC) that depended on dose and duration of exposure, following lifetime exposure to plasma concentrations relevant to clinical use [2]. Because rodent C cells express the GLP-1 receptor (GLP-1R) at much higher density than human C cells, and because no comparable signal has been clearly established in long-term human data, U.S. semaglutide labeling includes a boxed warning about thyroid C-cell tumors and contraindicates use in patients with a personal or family history of MTC or in patients with multiple endocrine neoplasia type 2 (MEN2), while noting explicitly that the relevance of the rodent finding to humans is unknown [3].

Papillary thyroid carcinoma (PTC) arises from follicular epithelial cells, a lineage distinct from the parafollicular C cells implicated in the rodent signal, and accounts for the majority of thyroid cancer diagnoses. A prior narrative review addressed GLP-1RAs and thyroid cancer broadly [4]. This review has a deliberately narrow scope. It asks what the literature shows about (i) semaglutide specifically; (ii) GLP-1RAs as a class, from which semaglutide-specific inferences can only be drawn indirectly; and (iii) PTC specifically, as distinct from differentiated thyroid cancer (DTC), MTC, and thyroid cancer overall. Because most human studies evaluated GLP-1RAs as a class and reported thyroid cancer without a histological breakdown, we state for every study how directly it addresses the semaglutide–PTC question (Table 1), and we do not attribute class-level or all-histology findings to semaglutide or to PTC.

## 2. Materials and Methods

### 2.1. Design

This is a narrative review intended to synthesize clinical, pharmacovigilance, receptor-expression, and preclinical evidence relevant to semaglutide, other GLP-1RAs, and PTC. No protocol was registered, and no PRISMA flow diagram was generated. This review was structured with reference to the SANRA (Scale for the Assessment of Narrative Review Articles) criteria [5] as a general standard of transparency for narrative reviews, without adopting a systematic-review protocol. A 2024 narrative review addressing GLP-1RAs and thyroid cancer broadly [4] is acknowledged as prior work that this review builds on and updates with more recent, PTC- and progression-specific data.

### 2.2. Search and Identification

PubMed (MEDLINE) was the primary bibliographic database and was searched between May and September 2026, with additional targeted searches conducted during peer review to evaluate studies and sources raised by reviewers. Searches combined terms for semaglutide and other GLP-1RAs with terms for thyroid neoplasia, including papillary, differentiated, and medullary thyroid carcinoma. Complementary searches addressed GLP-1 receptor expression in thyroid tissue and pharmacovigilance evidence (Supplementary Table S1). Google Scholar and publisher websites were used to retrieve full texts and to identify recently published or in-press studies, and the reference lists of relevant reviews, meta-analyses, and included studies were examined for additional primary reports. No publication-date restriction was applied, although particular attention was given to recent evidence through September 2026. Embase, Web of Science, and the Cochrane Library were not searched; this narrows the sensitivity of our search relative to a systematic review and is noted as a limitation.

### 2.3. Eligibility and Selection

Evidence was considered relevant when it reported thyroid-specific findings concerning semaglutide or another GLP-1RA and addressed thyroid cancer incidence, recurrence or progression, GLP-1 receptor expression in thyroid tissue, functional effects in thyroid carcinoma models, or thyroid cancer pharmacovigilance signals. Eligible designs were randomized-trial syntheses, cohort and case-control studies, target trial emulations, pharmacovigilance analyses, receptor-expression studies, preclinical studies, and case reports directly relevant to the question. Studies reporting cancer outcomes without a separable thyroid-specific result, duplicate publications, and editorials or commentaries without original data were not treated as primary evidence; commentaries were consulted only to contextualize the study they addressed. Titles, abstracts, and full texts were reviewed against these criteria by the authors. Because this was a narrative review, independent duplicate screening and a numerical study-flow log were not prospectively maintained, and this is acknowledged as a limitation.

### 2.4. Terminology and Evidence Labeling

“GLP-1RA” denotes the drug class; the dual GIP/GLP-1 agonist tirzepatide is included only where a study included it and is identified as such. “Semaglutide-specific” denotes an estimate derived from semaglutide exposure alone or from a semaglutide subgroup; “class-level” denotes an estimate derived from two or more GLP-1RAs, even where semaglutide was a component. “PTC” denotes papillary thyroid carcinoma; “DTC” denotes differentiated carcinoma of follicular-cell origin (papillary, follicular, and oncocytic), of which PTC is the most common; “MTC” denotes medullary thyroid carcinoma (C-cell origin); and “thyroid cancer overall” denotes analyses in which histologies were combined or not separately reported. These terms are not used interchangeably.

### 2.5. Evidence Appraisal

A single numerical risk-of-bias instrument was not applied because the review includes heterogeneous evidence types for which no single tool is appropriate. Each study was instead classified by drug specificity, endpoint specificity, and design, and was interpreted in light of the precision of its estimate, duration of exposure and follow-up, number of thyroid events, and design-specific sources of bias or confounding (Table 1). In descending order of inferential weight, we considered meta-analyses and pooled analyses of randomized trials; active-comparator cohort studies and target trial emulations; case-control studies; pharmacovigilance analyses; and case reports and preclinical studies.

**Table 1:** Summary of evidence and its directness to the semaglutide–PTC question.

Study [Ref.]	GLP-1RA Studied	Population/Design	Comparator	Follow-Up/Exposure	Thyroid Endpoint	Histology Reported?	Main Finding	Directness	Funding/Sponsorship
**A. Randomized-trial syntheses**									
Vilsbøll et al. 2026 [6]	Liraglutide, semaglutide (pooled)	93 phase 2/3 trials (6 CVOTs), 101,732 participants; postmarketing data; US claims. Manufacturer-sponsored.	Placebo; active comparators; SGLT2i (claims)	Trials 2006–2024; ≈207,000 patient-years	Thyroid cancer overall	No (thyroid cancer overall; PTC/MTC not separated)	HR 1.70 (0.99–3.03) vs. placebo; 1.83 (0.70–6.71) vs. active; CVOTs 1.41 (0.72–2.81); claims HR 0.87 (0.58–1.29) vs. SGLT2i. Postmarketing ≈ 0.001/100 patient-years.	Class-level (2 agents); thyroid cancer overall	Manufacturer-sponsored (Novo Nordisk); 6 authors are employees
Nagendra et al. 2023 [7]	Semaglutide	37 RCTs (16,839 placebo and 16,550 active-control patients) plus 19 real-world studies (13,330 patients)	Placebo; active comparators	RCTs ~24 weeks to 2 years (SUSTAIN 6 CVOT ~2 years); real-world studies 30 weeks to 18 months	Thyroid cancer overall	No (not subtyped in abstract)	OR 2.04 (0.33–12.61) vs. placebo; 1.19 (0.15–9.66) vs. active. Very wide CIs.	Semaglutide-specific; thyroid cancer overall	Not industry-funded (declared)
Feier et al. 2024 [2]	Semaglutide	10 RCTs; 14,550 participants (7830 on semaglutide)	Placebo or active comparator (varies by trial; RCTs from the semaglutide development program)	Trial-dependent	Thyroid cancer incidence	No (incidence reported as thyroid cancer overall, <1%)	Incidence <1%; no significant excess.	Semaglutide-specific; thyroid cancer overall	Not industry-funded (declared)
Silverii et al. 2024 [8]	GLP-1RAs (class)	64 RCTs of ≥52 weeks; 26 reported ≥1 thyroid cancer case	Any comparator	≥52 weeks; subset ≥104 weeks	Thyroid cancer overall; PTC and MTC subgroups	Yes (PTC, MTC)	Overall OR 1.52 (1.01–2.29), fragility index 1. ≥104 wk OR 1.76 (1.00–3.12). PTC OR 1.54 (0.77–3.06). MTC OR 1.44 (0.23–9.16).	Class-level; includes PTC-specific estimate	Italian Ministry of Health; IRCCS MultiMedica (non-industry)
Ko et al. 2026 [9]	GLP-1RAs and dual agonists	48 placebo-controlled RCTs, 94,245 participants; 14 cancer types assessed	Placebo	Median follow-up 70 weeks	Thyroid cancer (one of 14 cancer types)	No (thyroid cancer overall; 1 of 14 cancer types assessed)	OR 1.37 (0.82–2.31); 1 fewer to 9 more cases per 10,000; moderate certainty. Consistent in semaglutide- or tirzepatide-only analyses.	Class-level (semaglutide sensitivity analysis); thyroid cancer overall	Not industry-funded (declared)
Hu et al. 2022 [10]	GLP-1RAs (class)	45 RCTs; PROSPERO CRD42021289121	Placebo or other interventions	Trial-dependent (search to Oct 2021)	Thyroid cancer; thyroid disorders overall (6 outcomes)	No (thyroid cancer as one of 6 composite thyroid outcomes)	Thyroid cancer RR 1.30 (0.86–1.97), not significant. Overall thyroid disorders RR 1.28 (1.03–1.60), significant; driven by non-cancer diagnoses.	Class-level; thyroid cancer overall	Non-industry (declared)
Silverii et al. 2025 [11]	GLP-1RAs (class)	50 RCTs (≥52 weeks); overall and site-specific cancers	Any comparator	Trial-dependent (≥52 weeks)	Cancer overall; thyroid cancer as one of several site-specific cancers	No (thyroid cancer overall)	Overall cancer OR 1.05 (0.98–1.13), not significant. Thyroid cancer OR 1.55 (1.05–2.27), significant, more evident in longer trials. Colorectal cancer OR 1.27 (1.03–1.57), significant only in shorter trials. Uterine cancer reduced in obesity trials.	Class-level; includes thyroid-specific estimate	Italian Ministry of Health; IRCCS MultiMedica (non-industry)
Duchemin et al. 2025 [12]	GLP-1RAs (class; 9 agents including albiglutide, dulaglutide, efpeglenatide, exenatide, liraglutide, lixisenatide, semaglutide, taspoglutide)	PRISMA HARMS meta-analysis; PROSPERO CRD42024581184; 44 RCTs (from 5697 records screened, 361 eligible); 84,993 patients (84,156 with harms data); GLP-1RA arms 47,588 patients	Any control (placebo, active comparator, or mixed), pooled across trials	Trial-dependent; published manuscripts plus ClinicalTrials.gov aggregate data to 12 December 2024	Thyroid cancer (MTC, PTC, thyroid carcinoma, thyroid malignant, thyroid neoplasm, and thyroid tumor combined as one composite outcome)	No (histologic subtypes combined into one outcome)	Peto OR 1.62 (95% CI 1.08–2.44, I^2^ = 11%); 104 thyroid cancer cases identified (70 GLP-1RA arms, 34 control arms); incidence rate 0.5/1000 person-years (0.3–0.8); number needed to harm 1699. No significant subgroup effect by age, sex, BMI, follow-up, GLP-1RA type, or comparator (meta-regression). Authors concluded the association was “likely to be causal.”	Class-level; thyroid cancer overall (composite of multiple histologic terms)	Non-industry—“Review primarily funded by the authors’ own work”; no conflicts of interest declared
**B. Observational cohort, case-control, and target trial emulation studies**									
Bezin et al. 2023 [13]	GLP-1RAs (exenatide, liraglutide, semaglutide, albiglutide, dulaglutide)	French SNDS; T2D on second-line drugs 2006–2018; nested case-control; 2562 cases, 45,184 controls (matched for age, sex, diabetes duration)	Non-users; SGLT2i and DPP-4i analyzed separately	Cases 2014–2018; exposure ≤1, 1–3, >3 y; 6-month lag	Thyroid cancer overall; MTC	MTC yes (by diagnosis code); PTC not validated—subtype classification from claims codes was an acknowledged limitation [14]	Current use aHR 1.46 (1.23–1.74); 1–3 y 1.58 (1.27–1.95); >3 y 1.36 (1.05–1.74). MTC 1.78 (1.04–3.05) at 1–3 y. DPP-4i >3 y 1.19 (1.04–1.35).	Class-level; thyroid cancer overall and MTC	French national research funding (non-industry)
Morales et al. 2025 [15]	GLP-1RAs (albiglutide, dulaglutide, exenatide, liraglutide, lixisenatide, semaglutide; tirzepatide excluded)	T2D after metformin; new-user active-comparator cohorts; 10 claims and EHR databases (US, Germany, Spain, UK); only US cohorts passed diagnostics (51 of 120 comparisons)	SGLT2i, DPP-4i, sulfonylureas	ITT and on-treatment analyses; 1-year lag analysis; incidence 0.88–1.03 per 1000 person-years	Any thyroid tumor (benign and malignant); malignancy post hoc	No subtype; malignant vs. any only	Calibrated HRs 0.78–1.03 across comparators; none significant.	Class-level; thyroid tumors and malignancy	Non-industry (OHDSI network study)
Bea et al. 2024 [16]	GLP-1RAs (class)	Korean National Health Insurance claims; T2D; 21,722 GLP-1RA new users	SGLT2 inhibitors (the same paper separately analyzed DPP-4 inhibitor users)	Not stated in the accessible abstract (new-user design with propensity weighting)	Thyroid cancer	No (thyroid cancer overall)	HR 0.98 (0.62–1.53).	Class-level; thyroid cancer overall	Not reported
Pasternak et al. 2024 [17]	GLP-1RAs (liraglutide 57.3%, semaglutide 32.9%)	Denmark, Norway, Sweden 2007–2021; T2D; 145,410 initiators vs. 291,667 matched	DPP-4i (SGLT2i secondary)	Mean 3.9 y (SD 3.5); 25% >6 y	Thyroid cancer	Yes (PTC most common; subtype estimates imprecise)	HR 0.93 (0.66–1.31); MTC HR 1.19 (0.37–3.86); vs. SGLT2i HR 1.16 (0.65–2.05).	Class-level; thyroid cancer overall with subtypes	Swedish/Nordic research council funding (non-industry, declared)
Brito et al. 2025 [18]	GLP-1RAs (class)	US claims 2014–2021; T2D at moderate CV risk; target trial emulation; 41,112 GLP-1RA initiators (69 thyroid cancers)	SGLT2i, DPP-4i, sulfonylureas	Piecewise: <1, 1–2, ≥2 years after initiation	Incident thyroid cancer	No (could not separate MTC from other subtypes)	mITT HR 1.24 (0.88–1.76); first year 1.85 (1.11–3.08); as-treated 2.07 (1.10–3.95).	Class-level; thyroid cancer overall	Non-industry (Mayo Clinic/NIH-affiliated, declared)
Balachandra et al. 2025 [19]	GLP-1RAs (“GLP-1 analogs”)	Patients with thyroid nodules	Metformin	Not available from the accessible abstract	Thyroid cancer	Not available from the accessible abstract	RR 0.99.	Class-level; thyroid cancer in nodule population	Not reported
Sciscent et al. 2026 [20]	GLP-1RAs (class)	T2D 2017–2019; TriNetX; 5158–12,570 patients per matched cohort	SGLT2i, metformin, DPP-4i	5 years	Thyroid cancer	No (not subtyped in abstract)	RR 0.62 (0.37–1.05) vs. SGLT2i; 0.65 (0.32–1.31) vs. metformin; 0.91 (0.60–1.39) vs. DPP-4i.	Class-level; thyroid cancer overall	Not reported
Funch et al. 2021 [21]	Liraglutide	US commercially insured initiators	Metformin, exenatide, pioglitazone, DPP-4 inhibitors, or sulfonylureas	Median 17 months (US health plan, 2010–2014)	Thyroid cancer	Yes—85% papillary or follicular-variant PTC on medical record review; 46% were microcarcinomas ≤ 1 cm (67% in the liraglutide cohort vs. 43% in comparators)	RR 1.00 (0.56–1.79) vs. metformin to 1.70 (1.03–2.81) vs. all comparators excluding exenatide; latency-adjusted estimates slightly attenuated; no dose or duration effect.	Liraglutide-specific; thyroid cancer overall	Manufacturer-sponsored (Novo Nordisk); several authors are employees
Levy et al. 2025 [22]	GLP-1RAs pooled (206,844 pairs); semaglutide (100,494 pairs), liraglutide (28,629), dulaglutide (42,717)	Adults 18–75 with BMI ≥ 30; TriNetX 2013–2023; 1:1 propensity-matched	Untreated obesity	5 years	Thyroid and endocrine gland malignancies	No (PTC not isolated)	Pooled HR 0.70 (0.56–0.88); women 0.78 (0.61–0.99), men 0.65 (0.43–0.99). Semaglutide 0.54 (0.40–0.74); dulaglutide 0.96 (0.60–1.53); liraglutide 1.70 (1.03–2.82).	Drug-stratified (includes semaglutide); thyroid and endocrine gland cancers	Not reported
Baxter et al. 2025 [23]	GLP-1RAs (class)	Six population-based databases: Canada (Ontario), Denmark, Norway, South Korea, Sweden, Taiwan; T2D 2007–2023; 98,147 GLP-1RA users vs. 2,488,303 DPP-4i users	DPP-4 inhibitors	Median follow-up 1.8–3.0 years across sites (dose-timing analysis extended to 10 years)	Thyroid cancer	No (subgroup analysis by subtype not possible)	Pooled weighted HR 0.81 (0.59–1.12); fixed-effects. Random-effects HR 0.83 (0.55–1.27). No dose–response signal. 10-year cumulative rate ratio 1.00 (0.61–1.65) in supplementary analysis.	Class-level; thyroid cancer overall; largest and most geographically diverse cohort in this literature	Non-industry (multi-national academic/public-health consortium, declared)
**C. Progression in patients with existing thyroid cancer**									
Patrizio et al. 2026 [24]	GLP-1RAs incl. dual GIP/GLP-1 (tirzepatide)	536 exposed vs. 536 matched unexposed DTC; single institution; 84% AJCC stage I	Never exposed	Median follow-up 69 months; median exposure 16 months	Recurrence or structural progression	DTC, predominantly papillary	HR 0.87 (0.62–1.21) survival cohort; 0.75 (0.54–1.03) full cohort.	Class-level; DTC progression	Not reported
Patrizio et al. 2026 [25]	GLP-1RAs (semaglutide in 6 of 18)	18 patients (19 PTCs ≤ 1.5 cm) on active surveillance; single institution (same as above)	37 unexposed (38 PTCs), matched 1:2 by BMI and tumor size	Median follow-up 5.5 y; median exposure 25 months	Tumor growth kinetics	PTC only	Volume growth >72% in 2/19 vs. 1/38; not significant. Kinetics unchanged.	Class-level; low-risk PTC	Not reported
**D. Pharmacovigilance**									
Abi Zeid Daou et al. 2025 [26]	Several GLP-1RAs incl. semaglutide	FAERS 2004–Q1 2024; disproportionality	Other drugs in database	Not applicable	Thyroid cancer (reports)	No (not confirmable)	Semaglutide ROR 7.61 (6.37–9.08). Cannot yield incidence.	Semaglutide-specific; thyroid cancer overall	Not reported
Yang et al. 2022 [27]	GLP-1RAs (class)	FAERS 2004–2021	Other drugs in database	Not applicable	Tumor adverse events	No	Tumor-related signals for the class.	Class-level	Not reported
D’Arcy et al. 2026 [28]	GLP-1RAs (class)	FAERS retrospective analysis; 109,168 GLP-1RA monotherapy adverse-event reports (179 PTC, 77 MTC); negative binomial models; SEER population context	Overall FAERS proportions; adalimumab (PTC IRR); secukinumab (MTC IRR); SEER population context	2012–2021 database window; individual exposure duration, cumulative dose, and latency unavailable	PTC/MTC reporting proportions and modeled reporting-rate ratios	PTC and MTC report labels analyzed separately; no uniform histologic verification	RP: PTC 0.164% vs 0.0087% (18.9-fold); MTC 0.0705% vs. 0.0014% (50.7-fold), vs. overall FAERS. Author-reported IRRs (95% CI): PTC 18.17 (9.18–35.97) vs adalimumab; MTC 70.53 (8.45–588.98) vs. secukinumab (both *p* = 0.0001). Reporting signals, not population incidence.	Class-level; PTC- and MTC-specific reporting signals; not semaglutide-specific	Funding not reported in full text; authors declare no conflicts of interest
Anatriello et al. 2026 [29]	Semaglutide, liraglutide, exenatide, lixisenatide, dulaglutide, tirzepatide	34,956 individual case safety reports, EudraVigilance, January 2022–September 2024	Cross-drug disproportionality (ROR)	Not applicable	Thyroid cancer-related adverse events (reports)	No (not confirmable in spontaneous reports)	Semaglutide ROR 0.54 (0.37–0.81) vs. tirzepatide (lower reporting probability). Sensitivity analysis by indication: no significant signal for any individual agent.	Class-level (6 agents, drug-to-drug comparison); thyroid cancer overall	Not reported
**E. Preclinical studies**									
Wang et al. 2025 [30]	Semaglutide	PTC xenograft and macrophage co-culture	Vehicle-treated xenografts; untreated macrophage co-culture controls	Not applicable	Tumor growth; proliferation	PTC	No direct proliferative effect; reduced tumor size via M2-to-M1 macrophage reprogramming (GLP-1R/PPARG/ACSL1).	Semaglutide-specific; PTC-specific; preclinical	Not reported
Zhang et al. 2023 [31]	Liraglutide	GLP-1R-overexpressing papillary and medullary thyroid cancer cell lines	Untreated (vehicle) cell-line controls	Not applicable	Proliferation; migration	PTC and MTC lines	Inhibition via PI3K/Akt/mTOR-related signaling.	Liraglutide-specific; PTC and MTC; preclinical	Not reported
He et al. 2017 [32]	Liraglutide, exenatide	PTC tissue and cell models	Normal thyroid tissue (expression); untreated/vehicle-treated cells (functional assays)	Not applicable	Proliferation; Akt/Erk; energy metabolism	PTC	GLP-1R and IGF-1R expression higher in PTC than normal thyroid (49% of PTC tissues positive by IHC); liraglutide and exenatide exposure did not increase proliferation or change Akt/Erk signaling.	Class-level; PTC; preclinical	Not reported
**F. Case reports**									
Haddadin et al. 2026 [33]	Semaglutide	Single patient; metabolic syndrome	Not applicable	Not applicable	PTC diagnosis	PTC	Indolent presentation; no causal claim.	Semaglutide-specific; PTC; anecdotal	Not applicable (case report)
Beshay et al. 2026 [34]	Tirzepatide	Single patient	Not applicable	Not applicable	Mixed medullary–papillary carcinoma	Mixed MTC and PTC	Temporal association only.	Tirzepatide; MTC component; anecdotal	Not applicable (case report)
Stiewig Rapp and Basheer 2025 [35]	Semaglutide 0.25 mg weekly	35-year-old woman	Not applicable	Diagnosis 5 weeks after initiation	PTC, 6.8 cm	PTC	Likely pre-existing lesion; temporal association only.	Semaglutide-specific; PTC; anecdotal	Not applicable (case report)

Abbreviations: aHR, adjusted hazard ratio; AJCC, American Joint Committee on Cancer; BMI, body mass index; CI, confidence interval; CV, cardiovascular; CVOT, cardiovascular outcomes trial; DPP-4i, dipeptidyl peptidase-4 inhibitor; DTC, differentiated thyroid cancer; EHR, electronic health record; FAERS, FDA Adverse Event Reporting System; GIP, glucose-dependent insulinotropic polypeptide; GLP-1R, glucagon-like peptide-1 receptor; GLP-1RA, glucagon-like peptide-1 receptor agonist; HR, hazard ratio; IGF-1R, insulin-like growth factor 1 receptor; IHC, immunohistochemistry; IRR, incidence rate ratio; ITT, intention-to-treat; mITT, modified intention-to-treat; MTC, medullary thyroid carcinoma; NIH, National Institutes of Health; OHDSI, Observational Health Data Sciences and Informatics; OR, odds ratio; PTC, papillary thyroid carcinoma; RCT, randomized controlled trial; ROR, reporting odds ratio; RP, reporting proportion; RR, relative risk; SD, standard deviation; SEER, Surveillance, Epidemiology, and End Results; SGLT2i, sodium–glucose cotransporter 2 inhibitor; SNDS, Système National des Données de Santé; T2D, type 2 diabetes. “Directness” gives the drug level (semaglutide-specific or class-level) and the endpoint level.

## 3. Results

### 3.1. Biological Rationale and Receptor Expression Evidence

The regulatory warning on GLP-1RAs originates from rodent studies showing C-cell hyperplasia, adenomas, and carcinomas that depended on dose and duration of exposure after lifetime treatment, driven by dense GLP-1 receptor expression on rodent C cells. Human C cells express the receptor at substantially lower density, which is the main reason regulators have cautioned against direct extrapolation to humans.

Whether follicular cells, the cell of origin for PTC, express clinically meaningful levels of the GLP-1 receptor remains unsettled. Immunohistochemical studies have reported GLP-1 receptor immunoreactivity in a minority of PTC specimens [36,37], with expression in one study correlating with certain clinicopathologic features [37], whereas Waser et al. [38] found no expression in PTC and concluded that PTC is not a plausible target tissue for GLP-1RAs in humans. He et al. [32] found GLP-1R and insulin-like growth factor 1 receptor (IGF-1R) expression more pronounced in PTC than in normal thyroid tissue, yet GLP-1R agonist exposure did not increase PTC cell proliferation or measurably alter Akt/Erk signaling or cellular energy metabolism in their model. A polymorphism study linked a specific GLP-1R genetic variant and expression pattern to PTC risk in an Egyptian population [39].

The discordance among expression studies probably reflects methodology at least as much as biology. GLP-1R is a G-protein-coupled receptor for which antibody specificity has been a recognized problem: several commonly used antibodies have failed validation in receptor-null rodent tissue, and work with an extensively validated monoclonal antibody is the reference standard [40,41]. The thyroid studies differ in (i) reagent, (ii) detection principle (immunohistochemistry versus quantitative ligand-binding autoradiography), (iii) definition of positivity (intensity-only grading versus scored thresholds; cytoplasmic versus membranous), and (iv) sample size, which was small for PTC (17 cases in Gier et al. [36] and 56 in Jung and Kwon [37]). Gier et al. [36] reported immunoreactivity in 3 of 17 PTCs (18%); Jung and Kwon [37], who graded staining intensity alone, reported 18 of 56 PTCs (32.1%) with cytoplasmic immunoreactivity and negative normal follicular cells; and Waser et al. [38], who compared immunohistochemistry with quantitative autoradiography in 221 thyroid and pancreatic samples, found PTC negative by both methods. Ligand binding is not subject to antibody cross-reactivity, but each method has its own sensitivity limits, and detected receptor protein does not necessarily imply functional signaling; in the study that found higher expression in PTC, agonist exposure did not increase proliferation [32]. We therefore cannot determine from the published data whether GLP-1R is expressed in a reproducible subset of PTCs, and the studies are not directly comparable (Table 2).

Kennedy et al. [42] described GLP-1R overexpression as defining a distinct immunogenetic subset within primary and metastatic thyroid cancer. This observation is hypothesis-generating: it does not show that such tumors respond to GLP-1RA exposure, that they behave differently, or that the observation replicates, and no study we identified has stratified clinical outcomes by receptor status. If such a subgroup exists, population-level studies that do not stratify by receptor status could dilute a subgroup-specific effect in either direction.

### 3.2. Functional and Mechanistic Studies

Wang et al. [30] found that in PTC xenograft and macrophage co-culture models, semaglutide did not directly increase PTC cell proliferation but reduced tumor size by reprogramming tumor-associated macrophages from a protumor M2 phenotype toward an antitumor M1 phenotype, mediated through downregulation of PPARG via the GLP-1R/PPARG/ACSL1 pathway. This is a tumor-suppressive mechanism operating in the tumor microenvironment rather than directly on tumor cells. Zhang et al. [31] reported a related finding at the cellular level: liraglutide inhibited proliferation and migration of GLP-1R-overexpressing papillary and medullary thyroid cancer cell lines through changes in PI3K/Akt/mTOR-related signaling. Taken together, the available functional data, though limited to preclinical models, consistently point away from a proliferative effect of GLP-1RA exposure on PTC cells themselves, even in models selected for receptor expression.

### 3.3. Evidence from Randomized Controlled Trials and Large Pooled Trial Analyses

Directness: trial-level evidence is semaglutide-specific in [2,7] and class-level in [6,8,9] ([6] pools liraglutide and semaglutide); all report thyroid cancer overall, except [8], which also reports PTC and MTC separately. Vilsbøll et al. [6] pooled data from 93 liraglutide or semaglutide phase 2 and 3 clinical trials comprising 101,732 participants (about 207,000 patient-years), together with postmarketing and claims data. The analysis was sponsored by the manufacturer. Thyroid cancer events were few, and hazard ratios (HRs) were 1.70 (95% confidence interval [CI] 0.99–3.03) versus placebo and 1.83 (95% CI 0.70–6.71) versus active comparators, neither of which reached statistical significance. The authors noted that a trend toward more thyroid cancer diagnoses in GLP-1RA arms was plausibly explained by detection bias from more frequent clinical monitoring rather than a true excess in disease. Postmarketing surveillance in the same analysis found a thyroid cancer reporting rate of approximately 0.001 cases per 100 patient-years, although reporting rates cannot establish incidence.

Nagendra et al. [7], in a semaglutide-specific meta-analysis of 37 randomized controlled trials (RCTs) and 19 real-world studies, found no statistically significant difference in thyroid cancer overall versus placebo (odds ratio [OR] 2.04, 95% CI 0.33–12.61) or active comparators (OR 1.19, 95% CI 0.15–9.66); the intervals are wide enough to be compatible with either a large decrease or a large increase in risk. Feier et al. [2] systematically reviewed ten RCTs (14,550 participants, 7830 on semaglutide) and reported thyroid cancer incidence under 1% with no significant excess risk. Ko et al. [9] pooled 48 placebo-controlled trials of GLP-1RAs and dual agonists (94,245 participants; median follow-up 70 weeks) and reported a thyroid cancer OR of 1.37 (95% CI 0.82–2.31; moderate-certainty evidence), consistent in analyses restricted to semaglutide or tirzepatide. Silverii et al. [8] pooled 64 RCTs of at least 52 weeks and reported a modest increase in thyroid cancer overall (OR 1.52, 95% CI 1.01–2.29; fragility index 1), with non-significant subgroup estimates for PTC (OR 1.54, 95% CI 0.77–3.06) and MTC (OR 1.44, 95% CI 0.23–9.16). Two further randomized-trial meta-analyses reached different endpoints. Hu et al. [10], in a PROSPERO-registered meta-analysis of 45 trials assessing six thyroid outcomes, found no significant increase in thyroid cancer specifically (relative risk [RR] 1.30, 95% CI 0.86–1.97), while overall thyroid disorders as a composite outcome were significantly increased (RR 1.28, 95% CI 1.03–1.60), a signal the authors attributed to non-cancer diagnoses. Silverii et al. subsequently published a second, separate meta-analysis [11], distinct from their earlier thyroid-focused analysis [8], examining cancer risk broadly across 50 trials; it found no significant increase in cancer overall (OR 1.05, 95% CI 0.98–1.13) but did find a significant increase specifically in thyroid cancer (OR 1.55, 95% CI 1.05–2.27), more evident in longer-duration trials. Duchemin et al. [12], in a PRISMA HARMS-compliant meta-analysis prospectively registered on PROSPERO (CRD42024581184), combined published trial results with an aggregate analysis of ClinicalTrials.gov records across 44 RCTs (84,993 patients; 47,588 in GLP-1RA arms; 104 thyroid cancer cases identified in total, most not captured by earlier meta-analyses that relied on published reports alone). Using the Peto odds ratio, a method used for rare binary events, they found a statistically significant increase in thyroid cancer risk (OR 1.62, 95% CI 1.08–2.44, I^2^ = 11%), corresponding to an incidence rate of 0.5 cases per 1000 person-years and a number needed to harm of 1699; subgroup analyses and meta-regression found no significant influence of age, sex, body mass index, follow-up duration, GLP-1RA type, or comparator, and the authors concluded the association was “likely to be causal.” Three separate trial-level meta-analyses, spanning two research groups and overlapping but non-identical trial sets, have therefore each found a statistically significant increase in thyroid cancer of comparable magnitude (Peto OR 1.62; Mantel-Haenszel OR 1.52 and 1.55).

A recurring point, raised explicitly by Correra et al. [43] in an analysis of semaglutide’s oncogenic potential, is that most RCTs were not designed or powered to detect cancer endpoints. A null finding in this context indicates an absence of detectable excess risk within the trials’ statistical power, not evidence of oncological safety.

### 3.4. Evidence from Real-World Cohort and Case-Control Studies

Directness: all studies in this section are class-level, except Funch et al. [21], which is liraglutide-specific. The only drug-stratified analysis that includes semaglutide is Levy et al. [22] (Section 3.8), which reports thyroid and endocrine gland cancers combined. Bezin et al. [13] conducted a French nested case-control study using the national Système National des Données de Santé (SNDS) database and reported that one to three years of GLP-1RA use was associated with increased risk of thyroid cancer overall (adjusted HR 1.58, 95% CI 1.27–1.95) and MTC specifically (adjusted HR 1.78, 95% CI 1.04–3.05); the estimate beyond three years was lower (adjusted HR 1.36, 95% CI 1.05–1.74), and use of dipeptidyl peptidase-4 (DPP-4) inhibitors showed a weaker association (adjusted HR 1.19, 95% CI 1.04–1.35). This finding generated published discussion concerning residual confounding and detection bias [44,45,46], which the original authors addressed in a formal response [47].

Several larger and more recent cohort studies have not replicated this signal. Morales et al. [15] conducted a multi-database retrospective new-user cohort study using propensity score adjustment across United States and European claims databases. The primary outcome combined benign and malignant thyroid tumors, and only the U.S. cohorts (51 of 120 comparisons) passed the study’s prespecified diagnostics; in those analyses calibrated hazard ratios ranged from 0.78 to 1.03, and none was statistically significant. A nationwide Korean cohort study by Bea et al. [16] compared GLP-1RA users with sodium–glucose cotransporter 2 (SGLT2) inhibitor users (the same paper separately analyzed DPP-4 inhibitor users) and found no increased risk of thyroid cancer (HR 0.98, 95% CI 0.62–1.53). A Scandinavian cohort study spanning Denmark, Norway, and Sweden, with a mean follow-up of 3.9 years, similarly found no substantial increase in thyroid cancer risk (HR 0.93, 95% CI 0.66–1.31), with the upper confidence bound consistent with an increase of up to 31% [17]; semaglutide accounted for about one third of GLP-1RA use in this cohort. The largest and most geographically diverse cohort in this literature is Baxter et al. [23], which pooled six population-based databases from Canada (Ontario), Denmark, Norway, South Korea, Sweden, and Taiwan (98,147 GLP-1RA users and 2,488,303 DPP-4 inhibitor users with type 2 diabetes, 2007–2023). The pooled weighted hazard ratio for thyroid cancer was 0.81 (95% CI 0.59–1.12), with no increase in risk across cumulative dose categories; a supplementary analysis modeling time since initiation found no evidence of increased risk even at ten years (cumulative rate ratio 1.00, 95% CI 0.61–1.65), though this rested on fewer events than the primary comparison. Median follow-up ranged from 1.8 to 3.0 years across sites, and subgroup analysis by thyroid cancer subtype was not possible.

In a target trial emulation of United States claims data, Brito et al. [18] found no significant overall increase in thyroid cancer (HR 1.24, 95% CI 0.88–1.76) but a higher rate of diagnosis in the first year after initiation (HR 1.85, 95% CI 1.11–3.08); the as-treated estimate was also higher (HR 2.07, 95% CI 1.10–3.95). The authors suggested that earlier detection may explain this pattern but did not test it directly, and the study could not distinguish MTC from other thyroid cancer subtypes. Two additional recent cohort studies examined patients with thyroid nodules or broader comparator drug classes. Balachandra et al. [19] found no significant difference in thyroid cancer risk between GLP-1RA users (termed GLP-1 analogs by the authors) and metformin users among patients with known thyroid nodules (risk ratio 0.99), and Sciscent et al. [20], using the TriNetX database, found no increased five-year thyroid cancer risk with GLP-1RA use compared with SGLT2 inhibitors, metformin, or DPP-4 inhibitors. An earlier cohort study by Funch et al. [21], examining a United States commercially insured population initiating liraglutide (median follow-up 17 months), found relative risks ranging from 1.00 (95% CI 0.56–1.79) versus metformin to a significant 1.70 (95% CI 1.03–2.81) versus all comparators excluding exenatide; latency-adjusted estimates were attenuated. Medical record review found 85% of cases were papillary or a follicular variant of papillary carcinoma.

### 3.5. Progression in Patients with Existing Differentiated Thyroid Cancer

Directness: class-level exposure (all approved GLP-1RAs and dual agonists), DTC or PTC recurrence or progression, single institution. Most of the evidence above concerns new thyroid cancer in patients without known disease. Whether GLP-1RA exposure alters the course of an existing tumor is a separate question, and it has been examined in two cohorts from the same institution (Memorial Sloan Kettering Cancer Center), which are therefore not independent replications. Patrizio et al. [24] matched 536 patients with DTC who were exposed to any GLP-1RA or dual GIP/GLP-1 agonist, including tirzepatide, to 536 unexposed patients (1:1 by age, diagnosis date, stage, body mass index, and diabetes). The vast majority had papillary histology, and 84% had American Joint Committee on Cancer (AJCC) stage I disease. Over a median follow-up of 69 months, and with a median GLP-1RA exposure of only 16 months, exposure was not significantly associated with recurrence or progression (hazard ratio 0.87, 95% CI 0.62–1.21 in the survival cohort; 0.75, 95% CI 0.54–1.03 in the full cohort). The upper confidence limit in the survival cohort does not exclude a modest increase in hazard. The same group reported 18 patients with 19 low-risk papillary thyroid carcinomas (≤1.5 cm) under active surveillance who had received a GLP-1RA (semaglutide in 6 of 18) for a median of 25 months, matched 1:2 to 37 unexposed patients [25]. Significant volume growth occurred in 2 of 19 exposed and 1 of 38 unexposed tumors, without a statistically significant difference, and exposure did not change growth kinetics in the two exposed tumors that grew. Neither study evaluated semaglutide separately, and both are retrospective, single-institution, and limited by short drug exposure relative to the long natural history of DTC. Together, they show no statistically detectable adverse effect on progression at these exposure durations and sample sizes; they cannot establish that longer exposure, higher-risk disease, or semaglutide specifically is free of effect.

### 3.6. Evidence from Pharmacovigilance and Spontaneous Reporting Databases

Directness: a semaglutide-specific reporting odds ratio is available in [26] for thyroid cancer overall (histology not confirmable); [27] is class-level; [28] is class-level and reports PTC and MTC separately. Disproportionality analyses based on the U.S. Food and Drug Administration Adverse Event Reporting System (FAERS) produced a signal that differed from clinical trial and cohort data, with the caveat that such databases cannot establish incidence or causation. Abi Zeid Daou et al. [26] found statistically significantly elevated reporting odds ratios for thyroid cancer, for which histology could not be confirmed in spontaneous reports, for several GLP-1RAs, including semaglutide (reporting odds ratio [ROR] 7.61, 95% CI 6.37–9.08). Yang et al. [27], in an independent FAERS analysis spanning 2004 to 2021, similarly identified tumor-related adverse event signals associated with GLP-1RA use as a drug class. D’Arcy et al. [28] analyzed FAERS reports from 2012 to 2021, with Surveillance, Epidemiology, and End Results (SEER) incidence data used separately for population context. Among 109,168 GLP-1RA monotherapy adverse-event reports, they identified 179 PTC and 77 MTC reports. The PTC reporting proportion was 0.164%, compared with 0.0087% in the overall FAERS dataset, an 18.9-fold difference; the corresponding MTC proportions were 0.0705% and 0.0014%, a 50.7-fold difference. Negative binomial models yielded an author-reported incidence rate ratio (IRR) of 18.17 (95% CI 9.18–35.97) for PTC versus adalimumab and 70.53 (95% CI 8.45–588.98) for MTC versus secukinumab (*p* = 0.0001 for both). Despite the IRR terminology, these models used adverse-event reports as the denominator and therefore estimate relative reporting rates, not population-based cancer incidence. SEER and FAERS were not linked, and neither SEER context nor comparisons with other drug classes supplies the total number of exposed patients or eliminates differential reporting, surveillance bias, or confounding by indication. Individual exposure duration, cumulative dose, and latency were unavailable, and histologic labels were not uniformly verified. As with other FAERS-based findings discussed in this review, reporting patterns following publications and media coverage, along with the inability to confirm histologic subtype in many reports, limit how directly these signals can be applied to a PTC-specific question. A EudraVigilance disproportionality analysis [29] provides a European counterpart to these FAERS-based analyses, examining 34,956 individual case safety reports for semaglutide, liraglutide, exenatide, lixisenatide, dulaglutide, and tirzepatide between January 2022 and September 2024. Semaglutide had a significantly lower probability of reporting thyroid-cancer-related adverse events than tirzepatide (reporting odds ratio 0.54, 95% CI 0.37–0.81), and a sensitivity analysis stratified by therapeutic indication found no significant signal for any individual agent. Because this reporting system differs from FAERS in its incentives and media exposure, the absence of a clear class-wide signal here is consistent with, though it does not prove, the detection-bias explanation offered elsewhere in this review.

### 3.7. Case Reports

Case reports cannot inform incidence or causality; they are included only to document how the question has arisen in individual patients. Haddadin et al. [33] described PTC discovered during endocrine evaluation of a patient taking semaglutide for metabolic syndrome, with an indolent presentation. Beshay et al. [34] reported a mixed medullary–papillary carcinoma in a patient taking tirzepatide; because the tumor has a medullary component and the agent is a dual agonist, it bears on the MTC concern rather than on semaglutide and PTC. Stiewig Rapp and Basheer [35] described a 35-year-old woman diagnosed with a 6.8-cm PTC five weeks after starting semaglutide 0.25 mg weekly. A tumor of this size could not plausibly have arisen de novo in that interval; the case is therefore presented as a previously undetected lesion identified shortly after treatment initiation, that is, a temporal association and not evidence of accelerated growth or causation. We give these reports no inferential weight in the synthesis.

### 3.8. Sex as an Evidence Gap

PTC is more frequent in women, by a ratio of approximately three to one, and male sex is recognized as an adverse prognostic factor [48]. Thyroid cancer cells, including PTC, express estrogen receptors, and estradiol has been shown to promote proliferation in PTC cell lines [49], although epidemiologic studies have not established a clear association between thyroid cancer risk and female hormonal factors specifically. Whether sex modifies any association between GLP-1RA exposure and PTC has not been tested directly. Of the receptor studies reviewed, Jung and Kwon [37] examined sex as a correlate of GLP-1R expression in PTC and found no association; tumor multifocality was the only significant correlate, and it was inversely related to expression. Patrizio et al. [24] (71% female) and Bezin et al. [13] did not report sex-stratified estimates. The TriNetX analysis of 1.1 million patients with obesity found a lower five-year risk of thyroid and endocrine gland cancers in both sexes (HR 0.78, 95% CI 0.61–0.99 in women; 0.65, 95% CI 0.43–0.99 in men), but it compared treated patients with untreated patients, defined the outcome as thyroid and endocrine gland malignancy, and did not isolate PTC [22]. Sex-hormone receptor signaling has been studied in neuroendocrine neoplasms, including MTC, with variable and conflicting results across primary sites [50], but that literature does not address GLP-1RA exposure or PTC. Sex-stratified analysis is therefore an evidence gap: it is neither evidence of an effect nor evidence of no effect.

## 4. Discussion

The evidence reviewed includes a randomized-trial signal for thyroid cancer overall and non-significant cohort and progression estimates; interpretation depends on the uncertainties discussed below.

Reconciling these study types requires attention to what each design can and cannot show. Several semaglutide-specific syntheses and a large pooled trial analysis did not detect a statistically significant increase in thyroid cancer [2,6,7], whereas three separate class-level randomized-trial meta-analyses reported statistically significant increases in thyroid cancer overall [8,11,12]. Large cohort studies did not detect a statistically significant increase in new-onset thyroid cancer [15,16,17,23], and the progression cohort in patients with existing differentiated thyroid cancer found no statistically significant association over a median follow-up of 69 months, with a median drug exposure of only 16 months [24]. However, the generally indolent natural history of differentiated thyroid cancer makes duration of drug exposure an important limitation when interpreting these findings. Across the randomized and observational literature, median GLP-1RA exposure has generally not exceeded approximately four to five years, and even the Patrizio et al. cohort [24] paired a median of 69 months of surveillance with substantially shorter cumulative on-drug exposure. In the pooled analysis by Vilsbøll et al. [6], hazard ratios for thyroid cancer were directionally above unity across comparisons, including 1.70 versus placebo, with the lower confidence bound approaching the null, although none reached statistical significance. These estimates do not establish increased risk, but their direction and imprecision underscore the need for adequately powered studies with semaglutide exposure beyond five years and longer follow-up to determine whether a long-latency effect exists.

### 4.1. Drug-Specific Versus Class-Level Evidence

The regulatory concern arose from agent-specific rodent studies, yet nearly all human evidence on thyroid outcomes pools GLP-1RAs. Semaglutide accounted for about one third of GLP-1RA use in the largest Scandinavian cohort [17] and for 6 of 18 exposed patients in the active-surveillance cohort [25]; semaglutide-specific estimates come mainly from trial meta-analyses reporting thyroid cancer overall [2,7]. Class-level estimates cannot simply be assumed to apply to every agent and dose. In the TriNetX obesity cohort, estimates for thyroid and endocrine gland cancers versus untreated patients differed by agent: semaglutide HR 0.54 (95% CI 0.40–0.74), dulaglutide 0.96 (95% CI 0.60–1.53), and liraglutide 1.70 (95% CI 1.03–2.82) [22]. Comparison with untreated patients is vulnerable to confounding, and we do not read the semaglutide estimate as protective; the divergence across agents illustrates why class-level estimates cannot be assigned to semaglutide.

### 4.2. Histology-Specific Outcomes

Most estimates concern thyroid cancer overall. PTC-specific estimates include one trial meta-analysis (OR 1.54, 95% CI 0.77–3.06) [8] and subtype analyses in a Scandinavian cohort [17]. D’Arcy et al. [28] also reported a PTC-specific pharmacovigilance signal (Section 3.6), which cannot be interpreted as a population-based incidence estimate. The French case-control study reported MTC separately [13]. The progression evidence concerns DTC and PTC [24,25] but is not semaglutide-specific.

### 4.3. Exposure Duration and Latency

Differentiated thyroid cancer is typically indolent and can take years to become clinically apparent. Mean follow-up was 3.9 years in the Scandinavian cohort [17], median exposure was 16 and 25 months in the two progression cohorts [24,25], and median follow-up in the randomized evidence was only 70 weeks [9]. Follow-up therefore cannot exclude long-latency effects. Across the trial syntheses, pooled point estimates for thyroid cancer were above 1 (1.19 to 2.04) [6,7,9], with intervals that mostly included 1, and three separate meta-analyses of randomized trials have each reported a statistically significant increase in thyroid cancer overall (Peto OR 1.62, 95% CI 1.08–2.44 [12]; Mantel-Haenszel OR 1.52, 95% CI 1.01–2.29, fragility index 1 [8]; OR 1.55, 95% CI 1.05–2.27 [11]). These estimates warrant further study, but the overlapping trial sets mean that the three analyses cannot be considered independent replications.

### 4.4. Detection Bias and Confounding

Thyroid cancer diagnosis is sensitive to surveillance intensity, and obesity is itself associated with thyroid cancer risk. A meta-analysis of 21 articles reported 55% higher thyroid cancer risk in individuals with obesity than in normal-weight individuals, with a positive association also observed for PTC [51]. This association supports considering obesity as a potential confounder; it does not establish that obesity causes thyroid cancer. In the target trial emulation by Brito et al. [18], an excess of diagnoses in the first year after initiation (HR 1.85, 95% CI 1.11–3.08) coexisted with a non-significant overall estimate (HR 1.24, 95% CI 0.88–1.76) and a higher as-treated estimate (HR 2.07, 95% CI 1.10–3.95); the authors considered enhanced early detection a possible explanation but did not show that it accounts for the excess, and the study could not distinguish MTC from other subtypes. This pattern, and the confounding by indication that affects case-control and spontaneous-reporting designs [13,26,27], is consistent with detection bias but does not exclude a true effect, and the designs currently available cannot resolve the question.

### 4.5. Hierarchy of Designs and Convergence of Evidence

Randomized-trial syntheses provide the strongest inference but were not designed for thyroid endpoints; active-comparator cohorts and target trial emulations handle confounding better than case-control or spontaneous-reporting analyses, which are hypothesis-generating; case reports and preclinical studies cannot bear on incidence. A broader narrative appraisal of glucose-lowering therapies and cancer emphasizes the same limitations of trial-based cancer data (non-prespecified endpoints, limited oncologic adjudication, follow-up short relative to tumor latency, and low site-specific event counts) [52], and Ko et al. [9] report thyroid cancer separately (OR 1.37, 95% CI 0.82–2.31) within a broader synthesis of 14 cancer types. Mechanistic data do not resolve the question: receptor expression in PTC is inconsistently detected (Section 3.1), and the preclinical studies that tested functional responses did not show proliferative effects [30,31,32]. The evidence-gap analysis of Salama et al. [53] likewise identifies GLP-1RA effects on thyroid outcomes as requiring prospective study.

### 4.6. Regulatory Context and Clinical Translation

We note that Kennedy et al. [42] and D’Arcy et al. [28] share the majority of their authorship; the receptor-defined subgroup hypothesis and the SEER–FAERS reporting-proportion analysis of MTC and PTC therefore represent two related outputs of a single research group rather than independent lines of evidence, and should be weighted accordingly. The regulatory response to this question is informative in its own right. In the United States, semaglutide labeling carries a boxed warning about thyroid C-cell tumors and contraindicates use in patients with a personal or family history of MTC or in patients with MEN2; PTC or DTC alone is not a listed contraindication [3]. Canadian Ozempic labeling likewise includes an MTC/MEN2 contraindication and a serious warning about thyroid C-cell tumors [54]. Labeling is not uniform across jurisdictions: the U.K. Ozempic Summary of Product Characteristics lists hypersensitivity in Section 4.3 and discusses rodent thyroid C-cell tumors in Section 5.3 (preclinical safety data), without listing MTC or MEN2 as contraindications [55]. Clinical decisions should therefore follow the applicable agent-specific local labeling as well as professional guidance. In October 2023, the European Medicines Agency’s Pharmacovigilance Risk Assessment Committee (PRAC) reviewed exenatide, liraglutide, dulaglutide, semaglutide, and lixisenatide following a French signal [13] and concluded that the available evidence does not support a causal association between GLP-1RAs and thyroid cancer; no change to product information was recommended, and manufacturers were asked to continue monitoring [56]. A joint statement from the Society for Endocrinology, the British Thyroid Association, and the British Association of Endocrine and Thyroid Surgeons [57] reached a similar conclusion, explicitly citing both the Baxter et al. cohort and the PRAC decision. The statement notes that robust data on GLP-1 agonist safety in patients previously treated for thyroid cancer are limited, but that for differentiated thyroid cancer, including papillary, follicular, and oncocytic subtypes, GLP-1 agonists may be used alongside standard oncological surveillance where clinically indicated; that routine calcitonin measurement or thyroid ultrasound screening is not recommended in unselected patients; and that use should be avoided in patients with a history of medullary thyroid carcinoma, known pathogenic germline RET variants, or a family history of MEN2, concluding that the benefits of therapy are likely to outweigh any potential thyroid-related risks for most patients. Taken together, the available evidence and professional guidance suggest a provisional but specific answer to the question this review’s readership is most likely to ask: in a patient with treated papillary thyroid carcinoma or one under active surveillance for a low-risk tumor, and without a personal or family history of medullary thyroid carcinoma, a pathogenic RET variant, or MEN2, semaglutide or another GLP-1RA may reasonably be used alongside standard oncological surveillance; the progression cohort [24] and the active-surveillance cohort [25] provide direct, if limited, reassurance for this scenario. This should be understood as a provisional clinical judgment based on currently available evidence, consistent with existing professional guidance, rather than a guideline recommendation in its own right.

### 4.7. Limitations of the Review

In addition to the evidence-level limitations discussed above, this is a narrative review. PubMed was the primary database searched, so relevant studies indexed elsewhere may have been missed. Study selection and classification were performed without independent duplicate screening or a formal risk-of-bias instrument, so classification reflects the authors’ judgment, and the literature may have advanced after September 2026. Sex-stratified analyses and PTC-specific subgroup analyses were reported in too few studies to be synthesized (Section 3.8).

Several limitations recur across the body of evidence reviewed here. Follow-up duration and, importantly, cumulative GLP-1RA exposure remain short relative to the indolent natural history of most differentiated thyroid cancers. Adequately powered studies with semaglutide exposure beyond five years and longer follow-up will therefore be important to determine whether a long-latency effect exists. Observational and pharmacovigilance studies remain subject to detection bias. Obesity is a shared risk factor for both semaglutide use and thyroid cancer, which complicates adjustment in claims-based studies. Receptor expression studies use varied immunohistochemical methods and relatively small PTC samples, which may explain some of the disagreement among them. FAERS and other spontaneous reporting systems cannot generate incidence estimates. Sex-stratified analysis is reported in only a minority of the studies reviewed here, limiting the ability to draw conclusions about whether GLP-1RA-associated thyroid outcomes differ between men and women. This narrative review is also subject to the selection and interpretation limitations inherent to non-systematic methodology. A formal systematic review or meta-analysis restricted specifically to PTC, rather than thyroid cancer broadly, has not yet been published and would be a natural next step given the evidence gap identified by Salama et al. [53].

## 5. Conclusions

Within the sample sizes, exposure durations, and event numbers studied, most individual cohort studies, including the largest and most geographically diverse cohort identified [23], show no statistically detectable increase in thyroid cancer and no statistically significant association with progression of differentiated thyroid cancer in patients exposed to semaglutide or other GLP-1RAs. However, three separate meta-analyses of randomized trials, spanning two research groups and overlapping but non-identical trial sets, have each found a statistically significant increase in thyroid cancer overall (Peto OR 1.62, 95% CI 1.08–2.44 [12]; Mantel-Haenszel OR 1.52, 95% CI 1.01–2.29, fragility index 1 [8]; OR 1.55, 95% CI 1.05–2.27 [11]), with PTC- and MTC-specific subgroup estimates remaining non-significant. This consistency across analyses of overlapping randomized-trial evidence is not evidence of oncological safety and should not be dismissed as noise, even though its absolute magnitude is small (a number needed to harm of 1699 in the largest of the three analyses [12]) and cohort-level evidence has not reproduced it. Most trials and cohorts were not designed or powered for thyroid endpoints and report thyroid cancer overall; randomized evidence had a median follow-up ranging from about 70 weeks to several years across these analyses; population studies generally followed patients for less than five years; and the progression cohorts had median exposures of 16 to 25 months against a disease with a decades-long natural history [24,25]. Semaglutide-specific and PTC-specific evidence is sparse, and the progression data are derived from two non-independent, single-institution cohorts that evaluated GLP-1RAs as a class. Signals from a French case-control study, spontaneous-reporting analyses, and the first-year excess in one target trial emulation are compatible with detection bias and confounding by indication but cannot be dismissed on that basis alone. Receptor-expression findings are inconsistent, methods differ, and a receptor-defined, GLP-1RA-responsive PTC subgroup remains a hypothesis. Case reports do not inform causality, and sex-specific effects have not been studied.

Counseling of patients without a personal or family history of MTC or MEN2 should acknowledge the trial-level signal for thyroid cancer overall and the absence of a statistically significant association in the available cohort and progression studies, while explaining that long-latency, drug-specific, histology-specific, and receptor-status-specific effects have not been excluded. Priorities are semaglutide-specific, histology-resolved analyses with exposure and follow-up beyond five years; progression cohorts stratified by drug and receptor status; and sex-stratified reporting.

## Figures and Tables

**Table 2:** GLP-1R expression studies in PTC: methodological differences.

Study [Ref.]	Tissue and Sample	Detection Method	PTC Result	Comparability Notes
Gier et al. 2012 [36]	Human thyroid, including 17 PTCs, 15 normal thyroids, C-cell hyperplasia (9), MTC (12)	Immunofluorescence (dual-stain for calcitonin and GLP-1R); reagent not specified in the accessible abstract	3 of 17 (18%)	Study’s primary focus was C cells: GLP-1R detected in C cells (not follicular cells) in 5 of 15 normal thyroids (~35% of C cells assessed); PTC (17 cases) was a secondary comparison. Jung and Kwon separately found normal follicular cells negative.
Jung and Kwon 2014 [37]	56 PTCs, 4 MTCs, 7 nodular hyperplasias, 56 normal thyroids (postoperative specimens, single hospital)	Immunohistochemistry; intensity graded absent, weak, or strong (antibody manufacturer not specified in the accessible abstract)	18 of 56 (32.1%); cytoplasmic	Normal follicular cells negative. Multifocality inversely associated with expression; sex not associated.
Waser et al. 2015 [38]	221 combined human thyroid and pancreas samples (thyroid-specific subtype counts not given in the accessible abstract)	Immunohistochemistry with an extensively validated monoclonal antibody (MAb 3F52), compared with quantitative in vitro receptor autoradiography (and Western blot in selected cases) on the same samples	0 of 8 (0%); negative by both methods	Only study pairing IHC with quantitative autoradiography and Western blot validation. Sporadic MTC 2 of 10 (20%) positive; familial MEN2 MTC 0 of 10. Authors attribute Gier et al.’s higher estimates to insufficient antibody validation.
He et al. 2017 [32]	52 PTC and 55 normal thyroid tissue samples; IHH-4, BCPAP, and TPC-1 PTC cell lines compared with primary normal thyrocytes	Immunohistochemistry (Cell Signaling antibody; intensity × proportion score) and Western blot (Sigma antibody); MTT proliferation assay	49% positive by immunohistochemistry; normal thyroid samples negative	Liraglutide and exenatide exposure did not increase proliferation or alter Akt/Erk signaling despite higher expression.

Abbreviations: GLP-1R, glucagon-like peptide-1 receptor; IHC, immunohistochemistry; MAb, monoclonal antibody; MEN2, multiple endocrine neoplasia type 2; MTC, medullary thyroid carcinoma; MTT, 3-(4,5-dimethylthiazol-2-yl)-2,5-diphenyltetrazolium bromide; PTC, papillary thyroid carcinoma.

## Data Availability

No new data were created or analyzed in this study. Data sharing is not applicable to this article; all data discussed are available in the cited publications.

## Supplementary Materials

The following supporting information can be downloaded at: https://www.mdpi.com/article/10.3390/jcm15207834/s1, Table S1: Search strategy and search terms.
