# Supplementary material for: Semaglutide, Other GLP-1 Receptor Agonists, and Papillary Thyroid Carcinoma: A Narrative Review of Risk, Progression, and Mechanisms
Source: J Clin Med. 2026 Oct 11;15(20):7834. doi: 10.3390/jcm15207834 (PMC13704980; doi:10.3390/jcm15207834)
Supplement: Supplementary file 1 [file jcm-15-07834-s001.zip › jcm-4567500-supplementary.pdf]

Supplementary Table S1. Representative search strategies (PubMed/MEDLINE)

Searches were run between May and September 2026, with additional targeted searches conducted during peer review to evaluate studies and sources raised by reviewers. Record counts were not logged, consistent with the narrative (non-systematic) design of this review.

Strategy	Search string	Purpose
Strategy 1: GLP-1RA and thyroid neoplasia	(semaglutide[tiab] OR Ozempic[tiab] OR Wegovy[tiab] OR "glucagon-like peptide-1 receptor agonist*"[tiab] OR "GLP-1 receptor agonist*"[tiab] OR GLP-1RA*[tiab] OR liraglutide[tiab] OR dulaglutide[tiab] OR exenatide[tiab] OR tirzepatide[tiab]) AND ("Thyroid Neoplasms"[Mesh] OR "thyroid cancer"[tiab] OR "thyroid carcinoma"[tiab] OR "papillary thyroid"[tiab] OR "differentiated thyroid"[tiab] OR "medullary thyroid"[tiab])	Primary search for incidence, progression, and case reports
Strategy 2: Receptor expression	("GLP-1 receptor"[tiab] OR "glucagon-like peptide-1 receptor"[tiab]) AND thyroid[tiab] AND (expression[tiab] OR immunohistochemistry[tiab] OR immunoreactivity[tiab])	Receptor-expression and mechanistic studies
Strategy 3: Pharmacovigilance	(semaglutide[tiab] OR "GLP-1 receptor agonist*"[tiab]) AND (FAERS[tiab] OR "adverse event reporting system"[tiab] OR disproportionality[tiab]) AND thyroid[tiab]	Spontaneous-reporting analyses
